# Using non-exceedance probabilities of policy-relevant malaria prevalence thresholds to identify areas of low transmission in Somalia

**DOI:** 10.1186/s12936-018-2238-0

**Published:** 2018-02-20

**Authors:** Emanuele Giorgi, Ali Abdirahman Osman, Abdikarin Hussein Hassan, Abdi Abdillahi Ali, Faisa Ibrahim, Jamal G. H. Amran, Abdisalan M. Noor, Robert W. Snow

**Affiliations:** 1 Lancaster Medical School, Lancaster University, Lancaster, UK; 2 National Malaria Control Programme, Garowe, Puntland Somalia; 3 Ministry of Health, Hargeisa, Somaliland Somalia; 4 WHO Country Office, Hargeisa, Somaliland Somalia; 5 Population and Health Theme, Kenya Medical Research Institute-Wellcome Trust Research Programme, Nairobi, Kenya; 6 Centre for Tropical Medicine and Global Health, Nuffield Department of Clinical Medicine, University of Oxford, Oxford, UK

## Abstract

**Background:**

Countries planning malaria elimination must adapt from sustaining universal control to targeted intervention and surveillance. Decisions to make this transition require interpretable information, including malaria parasite survey data. As transmission declines, observed parasite prevalence becomes highly heterogeneous with most communities reporting estimates close to zero. Absolute estimates of prevalence become hard to interpret as a measure of transmission intensity and suitable statistical methods are required to handle uncertainty of area-wide predictions that are programmatically relevant.

**Methods:**

A spatio-temporal geostatistical binomial model for *Plasmodium falciparum* prevalence (*Pf*PR) was developed using data from cross-sectional surveys conducted in Somalia in 2005, 2007–2011 and 2014. The fitted model was then used to generate maps of non-exceedance probabilities, i.e. the predictive probability that the region-wide population-weighted average *Pf*PR for children between 2 and 10 years (*Pf*PR_2–10_) lies below 1 and 5%. A comparison was carried out with the decision-making outcomes from those of standard approaches that ignore uncertainty in prevalence estimates.

**Results:**

By 2010, most regions in Somalia were at least 70% likely to be below 5% *Pf*PR_2–10_ and, by 2014, 17 regions were below 5% *Pf*PR_2–10_ with a probability greater than 90%. Larger uncertainty is observed using a threshold of 1%. By 2011, only two regions were more than 90% likely of being < 1% *Pf*PR_2–10_ and, by 2014, only three regions showed such low level of uncertainty. The use of non-exceedance probabilities indicated that there was weak evidence to classify 10 out of the 18 regions as < 1% in 2014, when a greater than 90% non-exceedance probability was required.

**Conclusion:**

Unlike standard approaches, non-exceedance probabilities of spatially modelled *Pf*PR_2–10_ allow to quantify uncertainty of prevalence estimates in relation to policy relevant intervention thresholds, providing programmatically relevant metrics to make decisions on transitioning from sustained malaria control to strategies that encompass methods of malaria elimination.

**Electronic supplementary material:**

The online version of this article (10.1186/s12936-018-2238-0) contains supplementary material, which is available to authorized users.

## Background

All countries where malaria currently exists are encouraged to accelerate toward elimination [1, 2]. The pathway to elimination requires several important strategic policy decisions, adaptations of control interventions and changing malaria surveillance to become a key intervention [1, 2]. Many countries in Africa continue to support moderate-to-high *Plasmodium falciparum* transmission and must sustain funding to maintain high levels of vector control coverage, diagnostics and treatment. There are, however, large areas of Africa where the intensity of *P. falciparum* transmission has always been moderate-to-low or have transitioned to low transmission, in part as a direct result of intervention. These countries must decide how they might adapt previous control strategies that demand maintaining universal coverage to a more nuanced, cost-efficient and efficacious combination of interventions. For example, the inclusion of low dose primaquine to the standard artemisinin-based combination therapy for all clinical cases [3], the possible introduction of mass drug administration, with or without screening at detected foci of transmission [4], and the scaling back of universal coverage of insecticide-treated nets and intermittent presumptive treatment in pregnancy, toward more targeted interventions at foci of transmission [2, 5, 6].

During the Global Malaria Eradication era, recommendations were made to begin planning a pre-elimination stage when community-based parasite prevalence was consistently below 2% [7–9]. With time this included metrics based on the prevalence of infections in fevers below 5% [10]. The current international guidelines for malaria elimination remain non-specific on the precise criteria for accelerating elimination efforts, but define low transmission areas where community based prevalence is between 1–10% and very low as below 1% [2]. Less than 1% infection prevalence in the population has been identified as a signal for possible migration to an elimination-style approach for national malaria strategies [11]. However, it is recognized that there are areas which have recently transitioned to this state of parasite prevalence < 1%, while prevalence levels in sub-populations remain below a higher threshold (e.g., lower than 5% prevalence) suggesting heterogeneous endemicity. In these areas, immediate withdrawal of vector control is likely to result in rebound. This is distinguished from “low endemic malaria” where the natural state is such that transmission intrinsically occurs at a prevalence of < 1% because ecological conditions cannot support transmission above this value [11, 12].

For countries considering malaria elimination, or selecting sub-national areas to begin the process of elimination, knowing the epidemiological status of areas of low malaria prevalence, < 1% (very low) or < 5% (low), becomes an important pre-requisite. Deciding to change from national universal coverage of vector control and chemoprevention to focused targeting and increasing the sensitivity of surveillance systems comes with costs and benefits. To make definitions of low transmission operationally useful requires innovative analysis of field data to provide sensitive metrics at programmatically useful spatial resolutions to support malaria elimination activities.

Increasingly, model-based geo-statistical methods are applied to predict malaria prevalence across Africa at high spatial resolutions using temporally and spatially sparse empirical data [13, 14]. However, the uncertainty in relation to prevalence thresholds which define different control progress scenarios, has not been applied to national decision-making for malaria elimination. Estimates of prevalence are not sufficient to classify areas into different endemicity levels due to their intrinsic uncertainty. Current approaches used to convey uncertainty in estimates of mean predicted prevalence are based on the use of standard error or quantile (usually those at 0.025 and 0.975 levels) maps. These two approaches can help to visualize the overall dispersion around prevalence estimates but do not provide any information on the uncertainty relating to the exceedance, or not, of prevalence thresholds. This is particularly important in areas of low transmission, where heterogeneity increases and most survey data approach zero infection.

Here, a large collection of *P. falciparum* survey data from Somalia was used to demonstrate the value of summarizing prediction uncertainty with respect to policy-relevant prevalence thresholds in the sub-national targeting of pre-elimination, and formally introduce the empirical interpretation of uncertainty for decision-making.

## Methods

### Country and policy context

**Fig. 1:**
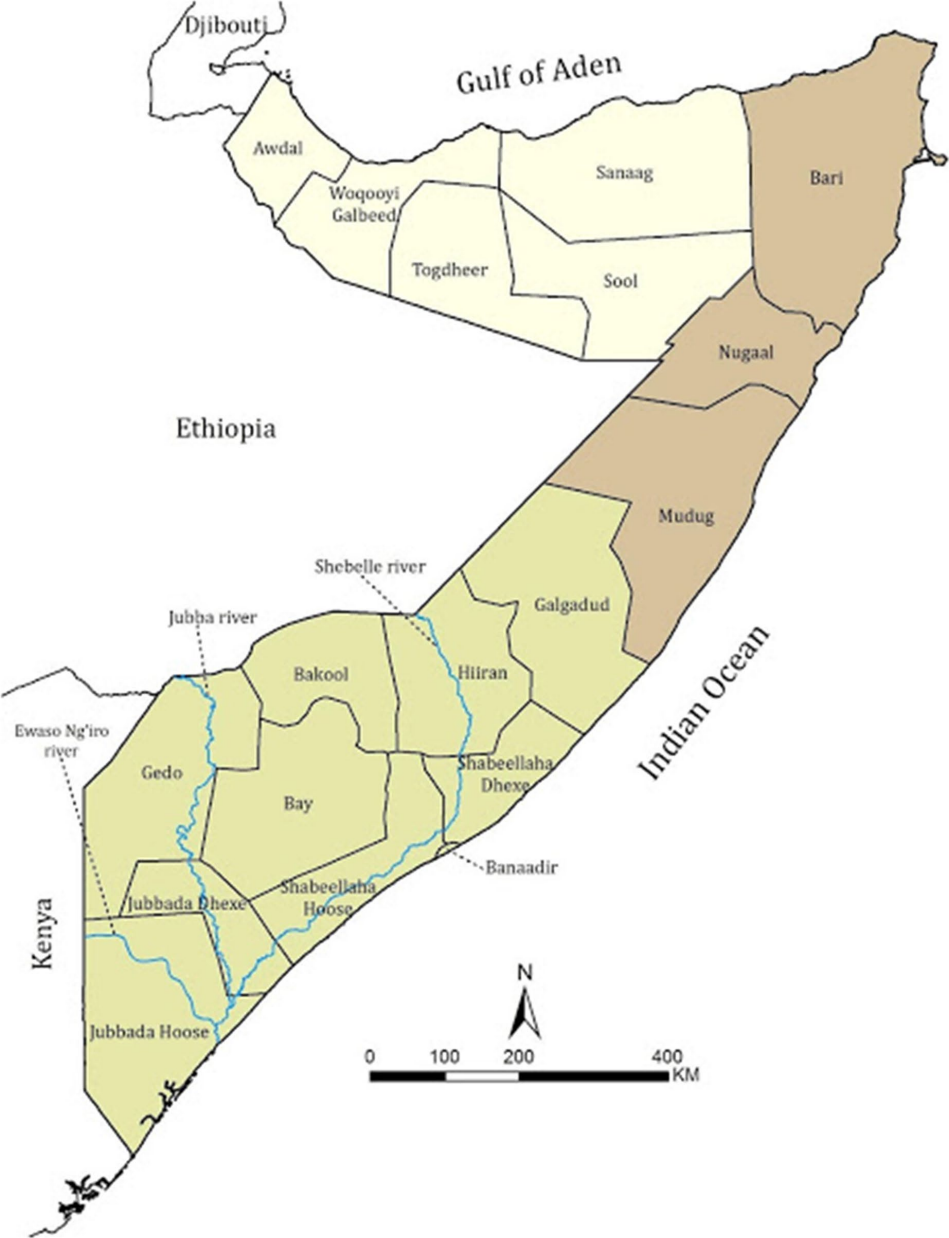
Somalia, showing three broad health regions of Somaliland (yellow), Puntland (brown) and South Central Zone (green) and the 18 health regions used to allocate federal resources

Somalia is in the Horn of Africa (Fig. 1) and has had a long history of sub-national targeting of vector control [15, 16] in concert with its extremely heterogeneous patterns of malaria transmission [17–19]. These ecologically driven heterogeneities have persisted over time [20, 21], however at the launch of the Roll Back Malaria initiative in 2000, Somalia elected to pursue a national strategy of universal coverage of insecticide treated nets (ITN) and presumptive treatment of all fevers with chloroquine [22], changing to artemisinin–sulfadoxine–pyrimethamine combinations in 2006 [23] and changed again in 2016 to artemether–lumefantrine [24]. The third national malaria strategic plan, 2011–2014, proposed a stratified approach emphasising the need for a targeted control effort to attain near zero transmission (parasite prevalence < 1%) in the north while maintaining sustained universal coverage of ITN in the southern regions [25]. The current 5-year malaria strategic plan, launched in 2017, is based on intervention strategies to sustain the prevalence levels below 1% in the north and increasing access to treatment and vector control in the south [26].

The country has three operational ministries of health, Somaliland, Puntland and the Federal government [27, 28]. The country is divided into 18 health regions, which serve as the principle sub-national levels of health information aggregation, management and resource allocation [27, 28] (Fig. 1).

### Population distribution

The last complete national census was undertaken during the 1970s. Using combinations of land cover, UN agency developed settlement databases and crude population counts at sub-national levels a modelled distribution and density of human settlement was developed for Somalia in 2010 [29]. This has recently been updated using data from a Population Estimation Sample Survey undertaken in 2013–2014 [30] and updated settlement, landcover and modelled approaches to distribution of populations [31]. Data were downloaded from WorldPop (http://www.worldpop.org.uk) at 1 decimal degree/1200 spatial resolution across each of the 18 health regions.

### Assembling parasite survey data

Sub-national and national cluster sample surveys of malaria intervention and infection prevalence were undertaken in 2005 [20, 21] and 2014 [32] respectively. Between 2007 and 2011, the Food and Agriculture Organization Food Security and Nutrition Analysis Unit (FAO-FSNAU) in Somalia, included finger prick blood samples for malaria testing using rapid diagnostic tests (RDTs) as part of routine monthly, sample nutritional surveillance across the country [20]. All community-based surveys included the examination of all residents within sampled households. Variations in age-groups per cluster were standardized to a single age group (see below). Each survey location was geo-coded to provide a unique longitude and latitude using a variety of methods and all checked using Google Earth. The final database included 2128 surveys at 1626 unique locations sampled between January 2005 and March 2014. Data were available for every region for each of the survey years; no data were available in 2006, 2012 and 2013. All surveys used RDTs for parasite detection, except in three locations where microscopy was used. For FSANU surveys ethical approval was provided by the Ministry of Health Somalia, Transitional Federal Government of Somalia Republic, Ref: MOH/WC/XA/146./07, dated 02/02/07 For the national survey in 2014 ethical approval was provided by the regional governments of Puntland (MOH/PL/DGO/196/013), Somaliland (MOH/DG/688/25001/13) and South Central Zone (MOHD&PS/DOH/00245/12/2013).

### Spatio-temporal geostatistical analysis

A spatio-temporal geostatistical model was developed to borrow strength of information in *Pf*PR between sampled locations and predict risk on a 1 by 1 km regular grid covering the whole of Somalia for every year 2005, 2007–2011 and 2014. Conditionally on a set of spatio-temporal random effects *W*(*x*,*t*), the counts of positive *P. falciparum* tests are assumed to follow mutually independent binomial distributions with number of trials *N*, corresponding to number of sampled individuals, and probability of a positive outcome *p*(*x*,*t*) at location *x* and year *t*, such that

1 $$\log\left\{\frac{p(x,t)}{1-p(x,t)}\right\} \; = \; \alpha \; + \; f\left( a \right)\; + \; g\left( A \right) \; + \; W\left( {x,t} \right)$$

where *f*(·) and *g*(·) are linear splines of the minimum and maximum age among the sampled individuals, denoted in the equation above by *a* and *A*, respectively. These were then used to standardize to a single age group 2–10 years (*Pf*PR_2–10_), traditionally used for malaria risk assessments [9, 33], when carrying out predictions, by setting *a* = *2* and *A* = *10*.

The spatio-temporal analysis of the data underwent several formative stages. First, a spatio-temporal exploratory analysis was undertaken, using a non-spatial binomial mixed model, where *W*(*x*,*t*) is assumed to be Gaussian noise, to assess the presence of residual spatio-temporal correlation in the data, based on the empirical spatio-temporal variogram (ESTV) of the point estimates of *W*(*x*,*t*). In a second step, a spatio-temporal covariance function was specified for the stochastic process *W*(*x*,*t*) [34] and fitted the model using Monte Carlo maximum likelihood. Finally, the validation of the model was carried out. Since the objective was to identify areas where prevalence lies below pre-specified thresholds, the validation approach aimed to test the validity of the adopted spatio-temporal structure for *W*(*x*,*t*). An algorithm was developed to generate 10,000 data-sets under the fitted model and, for each of these, the ESTV was computed as in the first step of exploratory analysis. The resulting 10,000 ESTVs were then used to generate 95% confidence intervals, at each spatial distance of the ESTV, under the assumption that the fitted model generated the data. If the observed ESTV fell within the 95% tolerance bandwidth, the conclusion was that there was no evidence against the adopted spatio-temporal covariance function. All computations were undertaken in the R software environment using the open-source package *PrevMap* [35]. Further details on the model formulation are provided in section 1.1 of Additional file 1.

### Non-exceedance probabilities of regional *Pf*PR_2–10_

For a given year *t* and region *R*, the target for prediction is the regional population-weighted average *Pf*PR_2–10_, formally defined as

$$p\left( {R,t} \right)\; = \; \frac{\sum_{x \in R} d(x) \hat{p}(x,t)
}{\sum_{x \in R} d(x)}$$

where the summations at the numerator and denominator are taken over a 1 km^2^ regular grid within region *R*,  ${\hat{p}}(x,t)$ is the predictor of *Pf*PR_2–10_ from the spatio-temporal geostatistical model and *d*(*x*) represents the density population extracted from the WorldPop database. Non-exceedance probabilities (NEPs) are used to summarize the uncertainty in the estimates of regional *Pf*PR_2–10_ with respect to their likelihood of being below a pre-defined threshold *l*. More specifically, NEPs are defined as the predictive probability that *Pf*PR_2–10_ is below *l*, formally expressed by NEP = Probability{*p*(*R*,*t*) < *l*|data}. Values of NEP close to 100%, indicate that *p*(*R*,*t*) is highly likely to be below the threshold *l*; conversely, for values close to 0%, *p*(*R*,*t*) is highly likely to be above *l*; finally, values around 50% correspond to the case of highest uncertainty, since *p*(*R*,*t*) is with equal probability below or above the threshold *l*.

## Results

The spatio-temporal exploratory analysis showed residual spatio-temporal correlation between data-locations, which mostly manifests within 2 years of time separation between surveys (Additional file 1). A spatio-temporal geostatistical model was fitted as specified by (1) in ‘Spatio-temporal geostatistical analysis’. The empirical spatio-temporal variogram fell within the 95% tolerance intervals generated by the algorithm described in SI-1.4 (Additional file 2). Hence, the conclusion was that the data do not show evidence against the fitted geostatistical model.

### Population-weighted mean prediction and standard error maps of *Pf*PR_2–10_

**Fig. 2:**
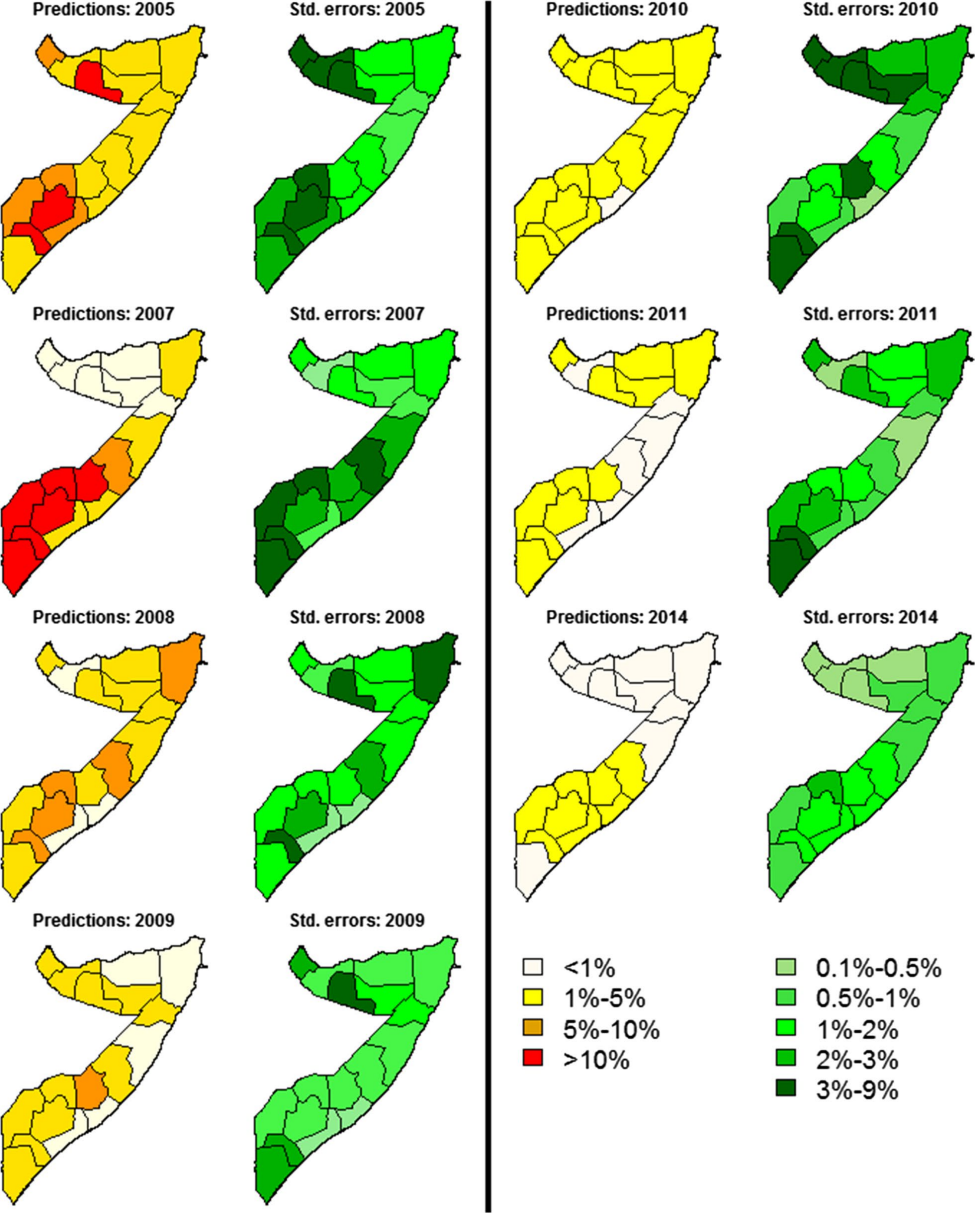
Mean predictions and standard errors for the regional population-weighted average *Pf*PR_2–10_ in the years 2005, 2007–2011 and 2014. The mean predictions are grouped into four classes: < 1, 1–5%, 5–10% and > 10%. The standard errors are grouped into six classes: 0.1–0.5%, 0.1–0.5%, 0.5–1%, 1–2%, 2–3% and 3–9%

Between 2005 and 2014, an overall decrease was observed in the mean predictions of the regional population-weighted average *Pf*PR_2–10_ (Fig. 2). However, two different sets of regions with distinct spatio-temporal patterns can be identified. The southern regions—with Glagadud being the northernmost—show a constantly decreasing trend in predictions but persistently higher than in the rest of Somalia, taking values below 1% only in Galgadud, Banaadir, Shabeellaha Hoose and Dhexe between 2008 and 2011. In the central and northern part of Somalia, the regional predictions show more variation over time, with values above 10% reached only in Togdheer in 2005 and between 5 and 10% in Awdal in 2005 and in Bari in 2008. All of the regions from 2011 onwards show predictions below 5%. In Fig. 2, the spatio-temporal variation in standard errors among the regions largely reflects that in the mean predictions, showing large values in standard errors associated with larger prevalence predictions. This can be noticed by the fact that all regions, except for Bay in 2007, that have predictions above 10% prevalence also have the highest standard errors between 3 and 9%. Additionally, in 2014, with all regions below 5%, the standard errors are between 2 and 3% in Bakool and below 2% in every other region. However, although the standard error maps can allow us to quantify the overall precision in regional estimates of prevalence, they do not provide any information in relation to the uncertainty of exceeding or not specific prevalence thresholds.

### Non-exceedance probability mapping to detect regions < 1 and < 5% *Pf*PR_2–10_

**Fig. 3:**
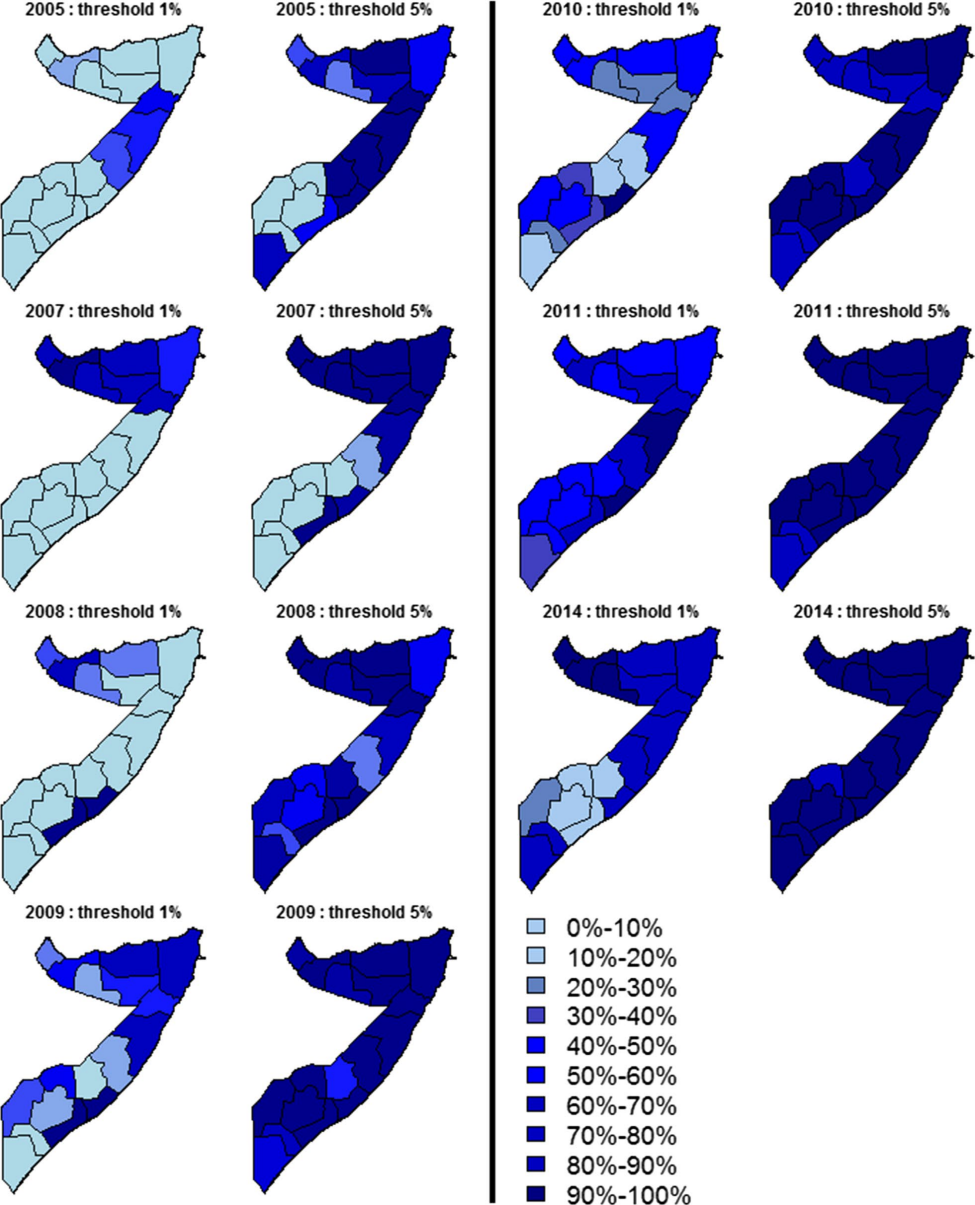
Maps of the non-exceedance probabilities for the regional population-weighted average *Pf*PR_2–10_ in the years 2005, 2007–2011 and 2014, with thresholds *l* = 1% and *l* = 5%. In the maps, the values are grouped into ten different classes, as indicated by the legend, from 0 to 10% of to 90–100%

The maps of NEPs the regional population-weighted average *Pf*PR_2–10_ show an overall increase between 2005 and 2014 in the probability that prevalence is below both 1 and 5% (Fig. 3). By 2010, all 18 regions in Somalia were confidently (> 70% likely) below 5% *Pf*PR_2–10_ and by 2014, there was a greater than 90% probability that 17 regions were below 5% *Pf*PR_2–10_ (Fig. 3) and between 80 and 90% probability in Bakool. The modelled data suggest that, by 2011, only two regions were more than 90% likely of being < 1% *Pf*PR_2–10_ and three different regions by 2014 but with most regions of Somaliland and Puntland being more than 70% likely of being < 1% *Pf*PR_2–10_. In 2014, Bakool, Bay, Hiiran and Shabeellaha Hoose, in the south, were more than 90% likely to be above a 1% threshold, whilst Awdal, Togdheer and Woqooyi Galbeed, in the north, were more than 90% likely to lie below 1%. For the remaining regions NEPs are between 20 and 90%. For any given year, comparison between the two NEPs maps, < 1 and < 5%, highlights that uncertainty with a respect to a threshold is not necessarily reflected in the other. For example, in 2005, Mugug and Nugal, in the central part of Somalia, had a probability between 40 and 60% of lying below 1%, but this becomes at least 90% if the threshold is 5%.

**Fig. 4:**
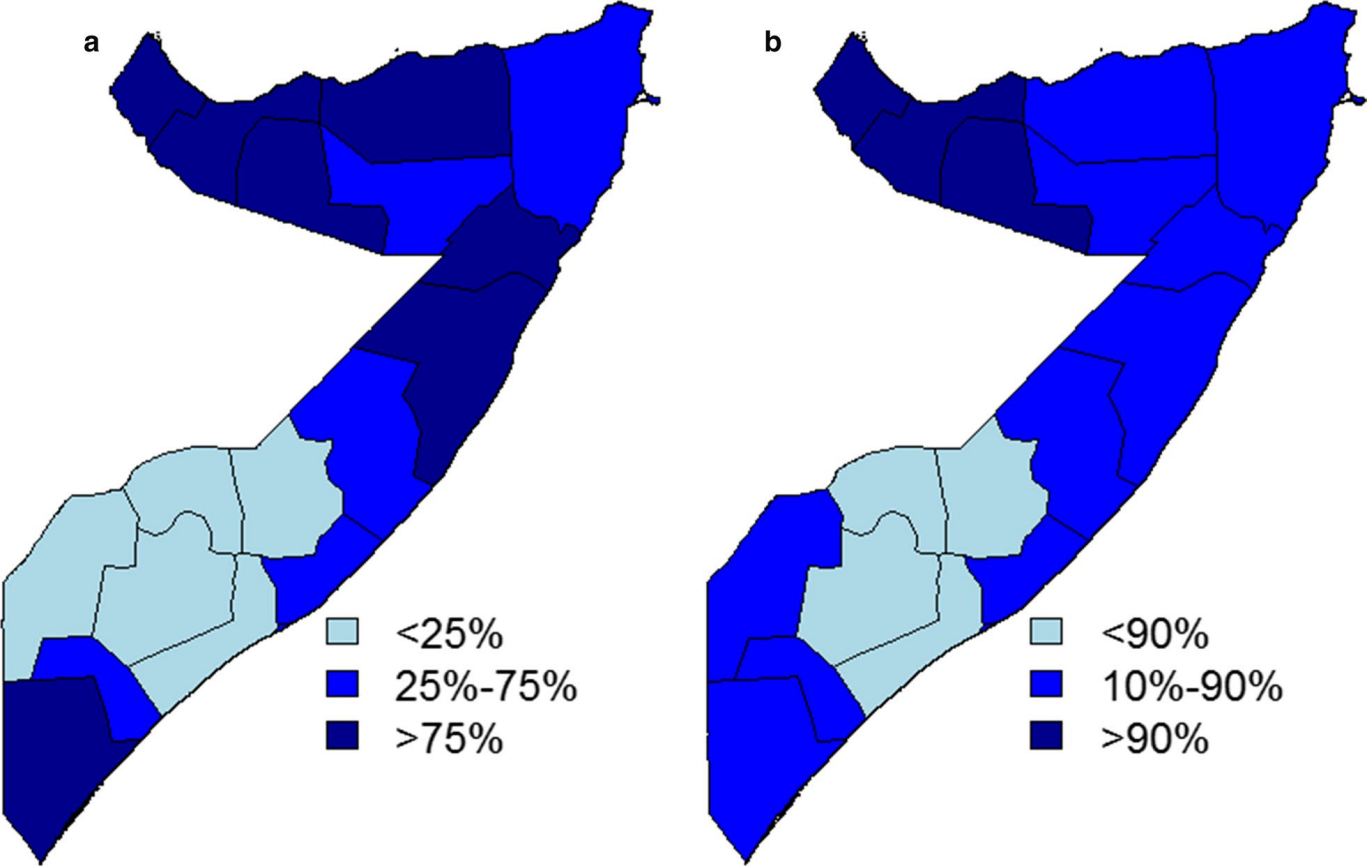
Maps of the non-exceedance probabilities for the regional population-weighted average *Pf*PR_2–10_ in 2014, using a *l* = 1% threshold. Each region is classified as < 25, 25–75% and > 75% in **a**, and as < 10, 10–90% and > 90% in **b**

Finally, for 2014, the standard approach was used to classify regions as < 1 or > 1% purely based on the mean predictions of prevalence (Fig. 3) and was compared with two other approaches that use NEPs (Fig. 4). In the first approach (Fig. 4a), a region was classified as < 1% if *Pf*PR_2–10_ is at least 75% likely to be below 1% or as > 1% if no more than 25% likely to be below 1%. A region was not classified if, instead, NEP is between 25 and 75%. In the second NEP approach (Fig. 4b), the rules for classification were made more stringent, defining a region as < 1% if NEP > 90%, as > 1% if NEP < 10% and as unclassified if 10% < NEP < 90%. The standard method which ignores the uncertainty in prevalence estimates classified all the central and northern regions, including Jubbasa Hoose in the south, as < 1% and all the remaining regions as > 1%. The first NEP approach based on the 25%/75% rule, instead, provided the same classification results for 14 regions but signalled that there was weak evidence (25% < NEP < 75%) to classify Jubbada Dhexe, Shabeellaha Dhexe and Galgadud, in the south, and Sool and Bari, in the north. As expected, the second NEP approach which requires even stronger evidence from the modelled data leaves more regions as unclassified, adding to the previous list two regions in the south and three in the north of Somalia.

## Discussion

Geostatistical methods provide a feasible and statistically principled approach to model spatio-temporally referenced survey data from low-resource settings [36]. One of the main advantages of these methods is that they also allow the estimation of risk at health decision making units and properties of uncertainty. To pursue this objective, the use of non-exceedance probabilities (NEPs) was proposed to quantify uncertainty in regional estimates of *Pf*PR_2–10_ with respect to policy relevant thresholds. Exceedance probabilities have been previously used to map malaria hotspots over continuous regions [37] but this is the first study that uses NEPs to reliably identify health decision making units with low levels of malaria transmission.

The spatio-temporal geostatistical analysis for Somalia suggested an overall decrease in *Pf*PR_2–10_ from 2005 to 2014, with all regions, except Bakool, showing more than 90% probability of *Pf*PR_2–10_ being < 5% by 2014. However, for 2014, the data provided weaker evidence in relation to a 1% threshold. Only the northern regions of Awdal, Woqooyi Galbeed and Togdheer showed a NEP no less than 90%. In the south, Shabeellaha Hoose, Bay, Bakool and Hiiran, instead, had a NEP < 10%, suggesting that these regions are most likely to have a *Pf*PR_2–10_ between 1 and 5%. However, in all remaining regions where NEPs were between 10 and 90%, inferences on *Pf*PR_2–10_ cannot be drawn with the same level of precision and, therefore, additional sampling effort would be required in these areas. All these aspects could not be discerned by the use of mean predictions and standard error maps.

The use of NEPS overcomes the limits of standard approaches that either are unsuitable to address policy questions based on thresholds interventions or incorrectly ignore the uncertainty in prevalence estimates. Standard error and quantile maps are an example of summaries of uncertainty that fail to address the specific public health issue: a large standard error or 95% prediction interval for prevalence do not provide any information on the uncertainty of exceeding or not a policy threshold. As a result, one of the most common, but incorrect, approaches is to use maps of prevalence predictions as a stand-alone tool to inform decision making, which can lead to the potential misclassification of health units as a consequence of ignoring uncertainty in prevalence estimates. The proposed approach instead can be used to plan future sampling efforts through the identification of regions where the mapped NEP does not reach acceptable levels (in the analysis two examples were given, where acceptable levels were set to 75 and 90% NEP) in order to make the best use of the available resources. This is particularly important in settings of low prevalence where sampling is often underpowered.

Other approaches that have been used to identify areas where prevalence exceeds a predefined threshold are based on *excursion sets* [38]. However, these are not defined when the target for prediction is an aerial average and have two main drawbacks with respect to NEPs: their definition is mathematically more complex and, therefore, more difficult to understand for policy-makers; they are exceedingly more conservatives than NEPs [36], i.e. areas with values of NEPs close to 100% might still be excluded from the resulting excursion set.

As malaria transmission in some countries declines, several have identified sub-national areas for malaria elimination, for example Djibouti, Sudan, Yemen, Pakistan and Afghanistan. In many settings, routine health information on fever malaria test positivity rates remains incomplete and rarely validated. Consequently, decisions are currently made on periodic national household sample survey data of infection prevalence. These surveys are often inadequately powered to detect very low levels of heterogeneous transmission. Nevertheless, model based geostatistical methods allows an interpolation of imperfect data in space and in time to provide properties of risk at administrative units required to make policy relevant decisions. However, this is only useful when decision makers can reliably interpret the level of uncertainty that underlies the predictions. The approach presented here provides a means to judge the probability of a health region falling in a category of risk that might define a transition from one strategic approach to another. This application of statistical modelling of uncertainty by exceedance probability has a wider value for other rare infections for which decisions between sustaining mass control and focused elimination are required sub-nationally. Additionally, the use of NEPs is not restricted to prevalence but can be extended to any disease risk metric that is estimated using a model-based approach. Neglected tropical diseases provide many examples where current intervention policies are defined in terms of exceedance of infection thresholds [39–41] and where the proposed approach in this paper would find a natural application.

## Additional files

**Additional file 1.** Model formulation and validation.

**Additional file 2.** Model outputs per region.
