# Supplementary material for: Using non-exceedance probabilities of policy-relevant malaria prevalence thresholds to identify areas of low transmission in Somalia
Source: Malar J. 2018 Feb 20;17:88. doi: 10.1186/s12936-018-2238-0 (PMC5819647; doi:10.1186/s12936-018-2238-0)
Supplement: Supplementary file 1 — Additional file 1. Model formulation and validation. [file 12936_2018_2238_MOESM1_ESM.docx]

**Additional file 1**

***1.1 Model formulation***

Let *Y_it_* denote the number of positive microscopy tests for *Plasmodium falciparum* out of *n_it_* in a community at location *x_i_* and year *t_i_*. We assume that conditionally on a Gaussian random effect, *W(x_i_,t_i_)*, the *Y_it_* are mutually independent Binomial variables with number of trials *n_it_* and probability of a positive microscopy test *p(x_i_,t_i_)* such that

$$\log\left\{ \frac{p(x_{i}, t_{i})}{1- p(x_{i}, t_{i})} \right\}= \alpha+f\left( a_{it} \right)+g(A_{it})+W\left( x_{i}, t_{i} \right)$$

where: *a_it_* and *A_it_* are the minimum and maximum age among the sampled individuals at location *x_i_* and year *t_i_*, respectively; *f* and *g* are linear splines, with knots at 3 and 10 years for *f* and at 80 years for *g*. We model the random effects as

$$W\left( x,t \right)=S\left( x,t \right)+Z\left( x,t \right)$$

where *S(x,t)* is a zero-mean stationary and isotropic spatio-temporal Gaussian process with variance σ^2^ and *Z(x,t)* is Gaussian noise with variance τ^2^. We interpret *S(x,t)* as the cumulative effect of unmeasured spatio-temporal risk factors for *P. falciparum* that accounts for extra-binomial variation between the sampled communities; *Z(x,t)*, instead, is an unstructured component that accounts for unexplained extra-binomial variation within communities (e.g. genetic variation).

We use a spatio-temporal correlation function of the Gneiting family [2], given by the following expression

$$cov\left\{ S\left( x,t \right),S\left( x^{'},t^{'} \right) \right\}=\frac{\sigma^{2}}{1+\frac{\left| t-t^{'} \right|}{\delta}} e^{-\left\| x-x^{'} \right\|/\varphi}$$

where *δ* and *φ* are scale parameters that regulates how fast the temporal and spatial correlations decay to 0, respectively.

Estimation of the model parameters is carried out using Monte Carlo maximum likelihood [1].

***1.2 The empirical spatio-temporal variogram***

Let $N(u,v)$ denote the set of observations such that $\left\| x_{i}-x_{j} \right\|=u$ and $\left| t_{i}-t_{j} \right|=v$. The empirical spatio-temporal variogram (ESTV) is defined as

$$\gamma\left( u,v \right)= \frac{1}{2\left| N(u,v) \right|}\sum_{\left( i,j \right)\in N(u,v)} \left\{ \tilde{W}\left( x_{i},t_{i} \right)-\tilde{W}(x_{j},t_{j}) \right\}^{2}$$

where$|N\left( u,v \right)|$ is the number of observations within $N(u,v)$ and $\tilde{W}\left( x,t \right)$ is a point estimate of$W(x,t)$ from a model that assume the absence of any extra-binomial spatio-temporal variation, i.e. *S(x,t)=0* for all *x* and *t*.

***1.3 Testing the presence of residual spatio-temporal correlation***

To justify the need of modelling the data using a geostatistical approach, we test the hypothesis on the presence of residual spatio-temporal correlation in the data. To pursue this objective, we then use the following iterative algorithm.

1. Obtain a point estimate of$W(x_{i},t_{i})$, say$\tilde{W}(x_{i},t_{i})$, at each of the observed locations *x_i_* and years *t_i_*, from a model that assume the absence of any residual spatio-temporal correlation, i.e. *S(x,t)=0* for all *x* and *t*.
2. Permute the order of the data, including$\tilde{W}(x_{i},t_{i})$, while holding $(x_{i},t_{i})$ fixed.
3. Compute the ESVM, as defined in the previous section.
4. Repeat 2 and 3 a large enough number of times, say *B*.
5. Use the resulting *B* empirical variograms to generate 95% tolerance intervals at each of the pre-defined distance bins.

If the ESVM obtained from the original, unpermuted data, lies outside these intervals, then the data show evidence of residual spatio-temporal correlation.

Figure SI-1 shows that the ESVM from the original data lies out the 95% tolerance intervals for time separations up to 2 years. However, the ESVM lies inside these intervals for time bins above 2 years, indicating a weak correlation between data points that are sampled more than 2 years apart.

***1.4 Model validation***

In order to check the validity of the chosen spatio-temporal covariance function, we modify the Monte Carlo algorithm introduced in the previous section by replacing step 2 with following.

2^*^. Simulate$W(x_{i},t_{i})$, at each of the observed locations *x_i_* and years t, from its marginal multivariate Gaussian distribution under the assumed model. Conditionally on the simulated values of$W(x_{i},t_{i})$, simulate binomial data *y_it_*, corresponding to the counts positive of positive microscopy tests. Finally, compute the point estimates $\tilde{W}(x_{i},t)$ using the simulated data.

In this case, the resulting 95% tolerance band of the ESTV is generated under the assumption that the true covariance function for$W(x_{i},t_{i})$ exactly corresponds to the one adopted in the analysis. If the ESVM obtained from the original data lies outside these intervals, then the data show evidence that the adopted spatio-temporal covariance function is not appropriate. If, instead, the ESVM from the data lies within the 95% tolerance band, we then conclude that the adopted covariance function is compatible with the data.

Figure SI-2 shows that the ESTV lies well within the 95% tolerance interval generated by the algorithm. This indicates that the data do not show evidence against the fitted spatio-temporal correlation function.


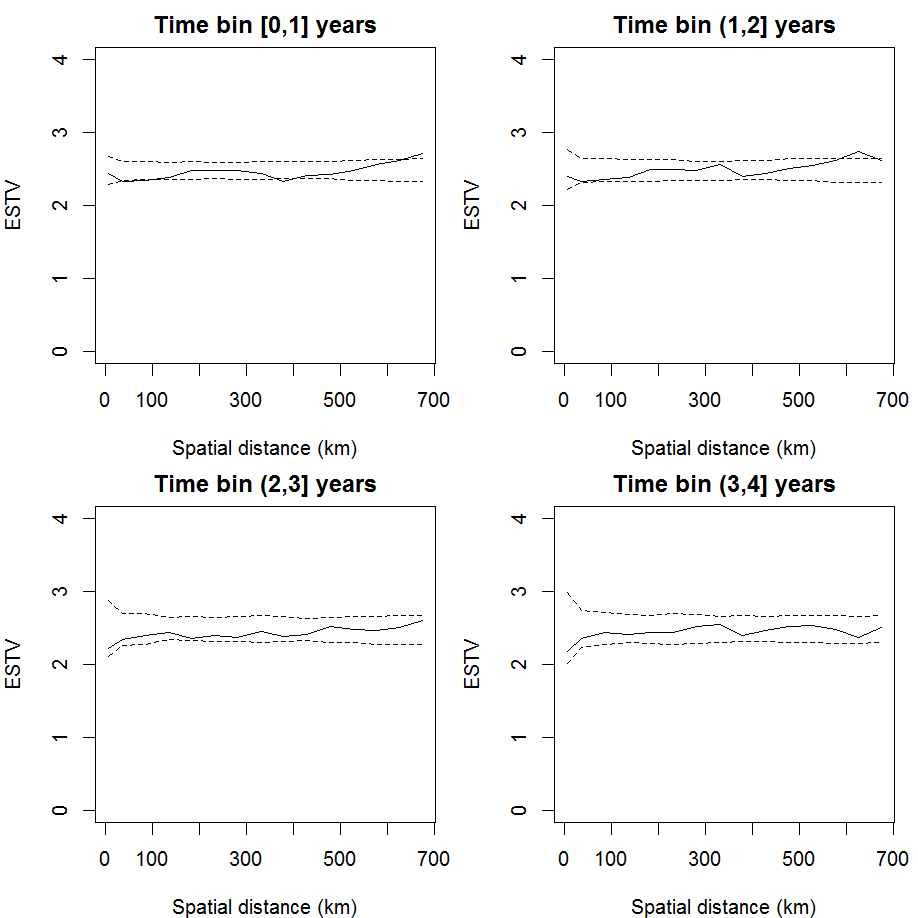


**Figure SI-1.** The solid line in each panel show the empirical spatio-temporal variogram (ESTV), defined in SI1, at four different time lags. The dashed lines represent the 95% confidence intervals generated under the hypothesis of spatio-temporal independence. At time lags of [0,1] and (1,2] years, the ESTV lies partly outside the 95% bandwidth, which is evidence of residual spatio-temporal correlation at the those time separations.


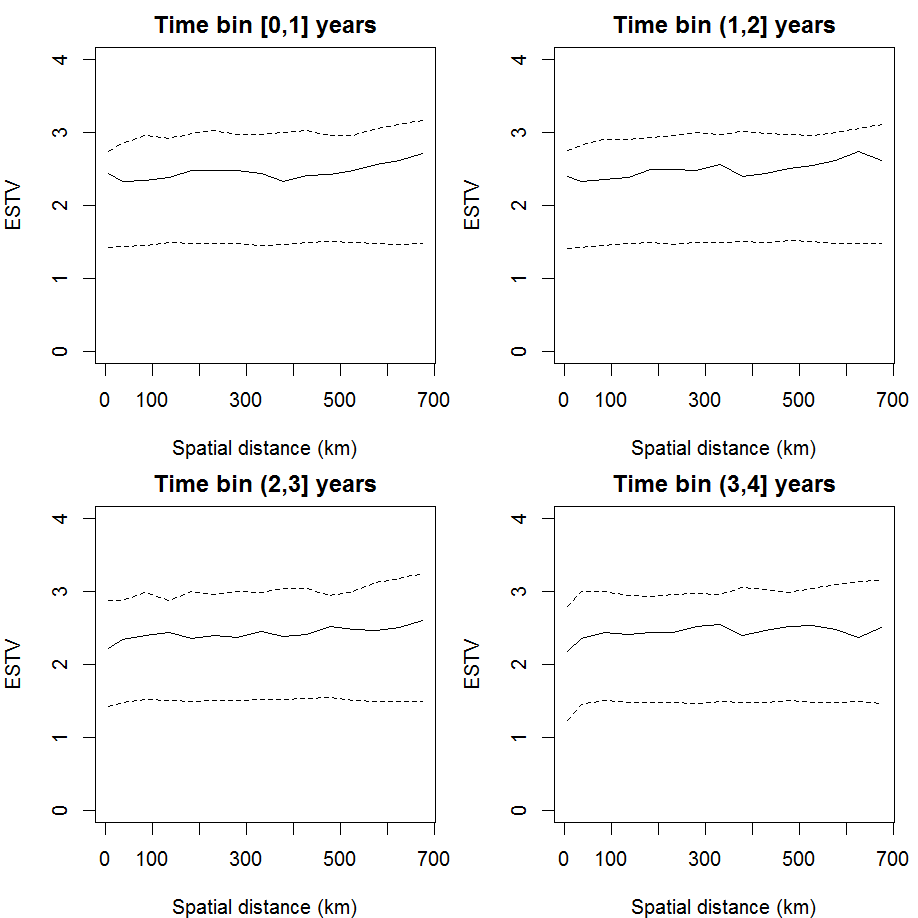


**Figure SI-2.** The solid line in each panel show the empirical spatial spatio-temporal variogram (ESTV), defined in SI1, at four different time lags. The dashed lines represent the 95% confidence intervals generated under the hypothesis that the fitted spatio-temporal covariance function correspond to the true covariance function that generated the data. At any of the four time lags, the ESTV falls within the 95% bandwidth, which is evidence that the adopted covariance function is compatible with the data.

**References**

1. Christensen OF (2004). Monte Carlo maximum likelihood in model-based geostatistics. *Journal of Computational and Graphical Statistics,* **13**: 702-718
2. Gneiting T (2002). Nonseparable, Stationary Covariance Functions for Space-Time Data. *Journal of the American Statistical Association,* **97**: 590-600
